# A novel method for the purification of inositol phosphates from biological samples reveals that no phytate is present in human plasma or urine

**DOI:** 10.1098/rsob.150014

**Published:** 2015-03-25

**Authors:** Miranda S. C. Wilson, Simon J. Bulley, Francesca Pisani, Robin F. Irvine, Adolfo Saiardi

**Affiliations:** 1 Medical Research Council Laboratory for Molecular Cell Biology, University College London, London, UK; 2 Department of Pharmacology, University of Cambridge, Tennis Court Road, Cambridge CB2 1PD, UK; 3 Department of Haematology, Cambridge University Hospitals NHS Trust, Hills Road, Cambridge CB2 0QQ, UK; 4 Department of Biology, Ecology and Earth Science, University of Calabria, Rende, Italy

**Keywords:** phytic acid, IP_6_, IP_7_, IP_8_, blood

## Abstract

Inositol phosphates are a large and diverse family of signalling molecules. While genetic studies have discovered important functions for them, the biochemistry behind these roles is often not fully characterized. A key obstacle in inositol phosphate research in mammalian cells has been the lack of straightforward techniques for their purification and analysis. Here we describe the ability of titanium dioxide (TiO_2_) beads to bind inositol phosphates. This discovery allowed the development of a new purification protocol that, coupled with gel analysis, permitted easy identification and quantification of InsP_6_ (phytate), its pyrophosphate derivatives InsP_7_ and InsP_8_, and the nucleotides ATP and GTP from cell or tissue extracts. Using this approach, InsP_6_, InsP_7_ and InsP_8_ were visualized in *Dictyostelium* extracts and a variety of mammalian cell lines and tissues, and the effects of metabolic perturbation on these were explored. TiO_2_ bead purification also enabled us to quantify InsP_6_ in human plasma and urine, which led to two distinct but related observations. Firstly, there is an active InsP_6_ phosphatase in human plasma, and secondly, InsP_6_ is undetectable in either fluid. These observations seriously question reports that InsP_6_ is present in human biofluids and the advisability of using InsP_6_ as a dietary supplement.

## 2. Background

Inositol is present in all eukaryotes, most archaea and some bacteria [1], but only nucleated cells have taken advantage of the metabolic stability of this sugar to evolve the complex array of phosphorylated signalling molecules known as inositol phosphates (if water soluble) or inositides (if lipid-bound) [2]. Attention has been drawn to the soluble inositol phosphates by the elucidation of the signalling pathway that connects receptor activation, via phospholipase C, to the release of the second messenger Ins(1,4,5)P_3_ [3]. A variety of inositol phosphates are also generated using Ins(1,4,5)P_3_ as precursor through a series of kinases and phosphatases [2]. Notably, the sequential action of the kinases IPMK (inositol polyphosphate multikinase, also known as Ipk2) [4] and IP_5_-2kinase (also known as IPPK or Ipk1) [5] convert Ins(1,4,5)P_3_ to InsP_6_ (inositol hexakisphosphate; phytic acid) [6,7]. InsP_6_ was originally discovered as a phosphate storage molecule in plant seeds but is now known to be present in all eukaryotic cells. It is the most abundant intracellular inositol phosphate species, with concentrations ranging between 10 and 50 µM in mammalian cells [8,9]. The social amoeba *Dictyostelium discoideum* has the highest known (non-plant seed) concentration, where InsP_6_ levels can reach 0.5 mM [8,10,11]. Biophysical studies have indicated that at cytoplasmic pH and salt concentrations, InsP_6_ exists in a neutral pentamagnesium form with a solubility limit of 49 μM [12], so it is likely that in *D. discoideum* some of the InsP_6_ is compartmentalized in vesicles, or that this organism has unusual cytoplasmic conditions permitting higher InsP_6_ solubility.

In mammals, were extracellular InsP_6_ to exist, it should only do so complexed to proteins, since it would be expected to precipitate in the prevailing salt and pH conditions. The suggested presence of InsP_6_ in human biofluids is currently relevant as ‘natural products’ companies are selling InsP_6_ as a supposedly beneficial dietary supplement. The claim is that dietary InsP_6_ is absorbed by the intestinal mucosa and transported in plasma, both of which assumptions have proved highly divisive. Specific InsP_6_ transporters are yet to be identified in mammals, and a molecule as polar as InsP_6_ cannot diffuse through the plasma membrane; thus its intestinal absorption is unlikely. By a variety of assays, the InsP_6_ concentration in human plasma has been calculated as 0.2–0.5 µM [13,14], but a more direct and highly specific mass assay was unable to confirm these values [8], showing that InsP_6_ could be present in human plasma at only sub-nanomolar concentrations, if at all.

Many signalling roles have been attributed to InsP_6_, notably a role in the control of nuclear–cytoplasm mRNA export [15]. The InsP_6_-derived inositol pyrophosphates have their own signalling roles and have been implicated in the pathophysiology of important human diseases such as diabetes, obesity, cancer, blood coagulation and viral infection (for reviews, see [16–18]). In these studies, the cellular biochemistry of InsP_6,_ InsP_7_ and InsP_8_ is often incompletely characterized, however. This is due to the technical difficulty of accurate measurement, which requires radioactive metabolic labelling and HPLC analysis [19]. These cumbersome techniques have held back InsP_6_ and inositol pyrophosphate research, which consequently lags far behind sister fields such as InsP_3_/Ca^2+^ and the inositol lipids [2]. The development of new methods to facilitate the analysis of InsP_6_ and inositol pyrophosphates is therefore imperative. A few years ago, a polyacrylamide gel electrophoresis (PAGE)-based method was developed for this purpose. This technique can easily resolve highly phosphorylated inositol species that are then visualized and quantified by staining with toluidine blue or DAPI [20,21]. This method has been well received as it does not require radioactive tracers and is now in common use for *in vitro* studies.

Because of the abundance of InsP_6_, InsP_7_ and InsP_8_ in *D. discoideum*, the PAGE technique can be used to study inositol pyrophosphate metabolism in this amoeba [10]. For mammalian extracts, however, the lower concentrations of inositol phosphates and thus larger extraction volumes have precluded direct analysis by PAGE. Here we describe a new inositol phosphate extraction method that overcomes these limitations, allowing the direct analysis of unlabelled InsP_6_ and inositol pyrophosphates extracted from mammalian cells and tissues. Since this newly developed technology allows the extraction of inositol phosphates from large sample volumes, we also used it to test for the presence of InsP_6_ in human biofluids in an attempt to resolve the controversy [8,22,23] surrounding this issue.

## 3. Material and methods

### 3.1. Cell culture and treatment

Mammalian, plant and fly cells used were gifts from several different laboratories and were grown in standard conditions for each cell type. HCT116 cells were cultured in DMEM (Invitrogen) supplemented with 10% FBS, in 5% CO_2_. Vegetative state *Dictyostelium discoideum* cells were grown at 22°C in a shaking HL5 medium supplemented with 100 U ml^−1^ penicillin and 100 μg ml^−1^ streptomycin (Invitrogen). For sodium fluoride treatment, 90% confluent HeLa, MCF7 and HCT116 cells (2× 14 cm dishes) were treated with 10 mM sodium fluoride (Sigma) for 1 h before harvesting by trypsinization. For oligomycin treatment, the cells were pre-treated with glucose-free DMEM for 30 min, before addition of 5 μM oligomycin (Sigma) for 3 h. Cells were harvested by trypsinization.

### 3.2. Titanium dioxide bead extraction

All steps in the extraction until elution were performed at 4°C to avoid acid degradation of inositol pyrophosphates. First, the titanium dioxide (TiO_2_) beads (Titansphere TiO 5 µm; GL Sciences) were weighed and prepared by washing once in water then once in 1 M perchloric acid (PA). Generally 4–5 mg of beads was used for each sample. After centrifuging at 3500*g* for 1 min, the beads were resuspended in PA.

Cells were harvested as appropriate and washed in PBS. A small aliquot was removed for later protein quantification, enabling normalization. The cells were pelleted and extracted using 800 µl PA (pH 1). After resuspension in the acid, samples were kept on ice with vortexing for 10 min, then centrifuged at 18 000*g* for 5 min, at 4°C. The supernatants were removed into new eppendorfs and TiO_2_ beads added (4 mg in 50 μl PA). Samples were vortexed briefly then rotated at 4°C for 15 min; the inositol phosphates and other molecules were adsorbed onto the beads at this point. Beads were pelleted by centrifuging at 3500*g* for 1 min, and then washed twice in PA with supernatants discarded. To elute, 200 µl 10% ammonium hydroxide (pH 10) was added to the beads. Samples were vortexed briefly before rotation for 5 min. After centrifuging, the supernatants (containing the inositol phosphates) were transferred into new eppendorfs. The elution procedure was repeated on the beads to ensure full recovery, and the second supernatants added to the first. The samples were then vacuum evaporated to 50 µl for PAGE or other further analysis. Alternatively, samples were evaporated until at pH 7 then stored at 4°C or −20°C.

The protocol used for TiO_2_ extraction from *Dictyostelium* PA extracts, diluted InsP_6_ standards and radioactive ^3^H-Ins(1,4,5)P_3_ (PerkinElmer) or ^3^H-InsP_6_ (Amersham) was the same as above, except that the standards were directly added to 1 ml PA. For the radioactive experiments, 5 ml of Ultima Gold (PerkinElmer) scintillation cocktail was added to the TiO_2_ eluate and the samples were counted in a β-counter.

Mouse liver and brain were collected from newborn (P1) pups and rapidly frozen. PA (2 ml) was added to approximately 0.5 g of tissues, equivalent to one liver or two brains. The organs were rapidly homogenized in an electric blender and incubated in ice for 10 min. The samples were centrifuged at more than 15 000*g* for 15 min and the supernatant used for TiO_2_ bead extraction.

### 3.3. Plasma, serum and urine extraction

Bovine and horse serum and plasma were bought from Life Technologies and Sigma Aldrich. Human serum and plasma were bought from TCS Biosciences and Sigma Aldrich. Alternatively, human plasma was prepared from anonymous donors. Two 20 ml samples of blood from each volunteer were collected into tubes pre-filled with 1.6 mg of EDTA per ml of blood (5.5 mM) and immediately cooled on ice for 10 min. One of the samples was then spiked with 1 nmol of InsP_6_ prior to removal of cells and platelets by centrifugation at 1500*g* for 10 min at 4°C. This yielded plasma for analysis. To extract InsP_6_, half volume of 2 M PA was added to 10–20 ml of serum or plasma and the sample rotated at 4°C for 30 min. The denatured proteins were removed by centrifugation at 15 000*g* at 4°C for 30 min. The supernatant was subjected to the extraction procedure as described above, using 5 mg of TiO_2_ beads.

Human urine was obtained from anonymous donors. After centrifugation at 2000*g* at 4°C for 5 min to remove any epithelial cells, which were discarded, the samples were split in half; one half was spiked with 2 nmol InsP_6_. Concentrated PA was added to a final concentration of 1 M and rotated at 4°C for 30 min. The denatured proteins were removed by centrifugation at 15 000*g* at 4°C for 30 min. The supernatant was subjected to the extraction procedure as described above.

### 3.4. Enzymatic treatment

Cell extracts were treated with apyrase (New England Biolabs) following the manufacturer's instructions.

### 3.5. Polyacrylamide gel electrophoresis of inositol phosphates

PAGE was performed as previously described [20]. Briefly, 35% polyacrylamide/TBE gels were used to resolve the TiO_2_-extracted samples. Samples were mixed with either orange G or bromophenol blue loading buffers. The gels were pre-run for 30 min at 300 V and run overnight at 4°C at 600 V and 6 mA, until the orange G had run through two-thirds of the gel. Gels were stained as previously described [20] with either DAPI or toluidine blue. Gels stained with toluidine blue were scanned using a desktop computer scanner for image analysis. ImageJ was used for densitometry (*n* = 3 per experiment), and amounts of inositol pyrophosphates are expressed as a ratio of their band density over InsP_6_. Nucleotides and polyP standards were bought from Sigma Aldrich, while InsP_6_, InsP_5_, InsP_4_ and InsP_3_ were bought from Sichem.

## 4. Results

### 4.1. Titanium dioxide binds to inositol phosphates

**Figure 1.**
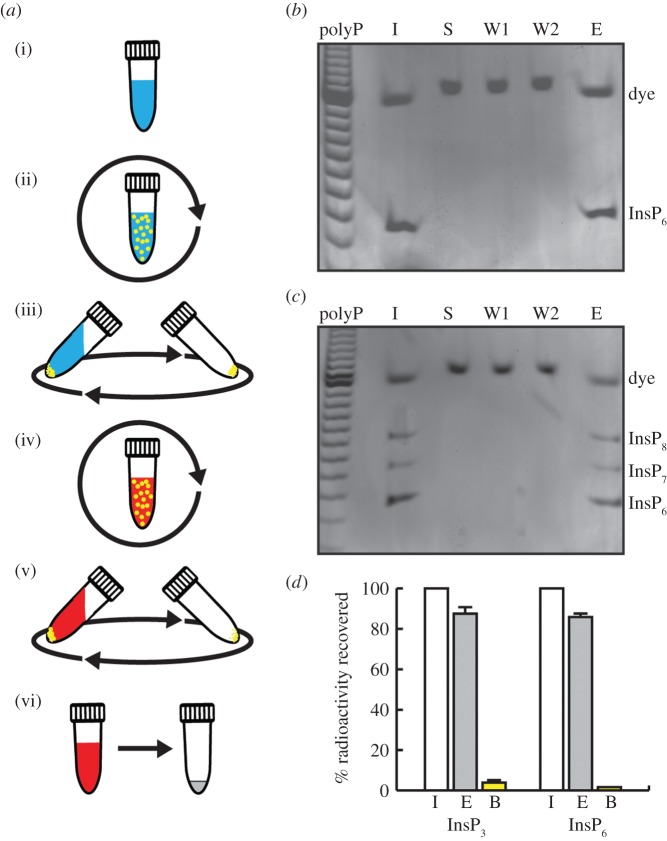
TiO_2_ purifies inositol phosphates. (*a*) Flowchart describing the five-step TiO_2_ bead extraction procedure. (i) Acidic solution (blue) containing inositol phosphates is incubated with (ii) TiO_2_ beads (yellow) for 10 min, before (iii) spinning and washing the beads twice with 1 M PA. Elution occurs by incubation (iv) at basic pH (red) with subsequent spinning and recovering the supernatant (v). This is evaporated (vi) to concentrate and neutralize (grey) the extract. (*b*) InsP_6_ diluted in 1 M PA was purified using TiO_2_ and subjected to PAGE with toluidine blue staining. I, input; S, supernatant; W1 and W2, washes; E, eluted. While all the eluted InsP_6_ was loaded on the gel only 1/10 (approx. 100 µl) of the S, W1 and W2 fractions were loaded. The acid in these fractions results in slightly compressed and slower migration of the orange G dye. (*c*) As (*b*), but using a PA extraction from vegetative state *D. discoideum* cells as input. These toluidine blue-stained gels are representative of experiments performed at least three times. (*d*) To calculate the exact percentage of recovery, radioactive ^3^H-Ins(1,4,5)P_3_ and ^3^H-InsP_6_ were subjected to TiO_2_ purification. The radioactivity recovered (E) and radioactivity remaining on TiO_2_ beads (B) were normalized to the respective radioactive input (I). The graph showing the average ± s.d. (*n* = 4) is representative of two independent experiments with matching results.

The ability of titanium dioxide (TiO_2_) to bind with very high affinity to phosphate groups is used in phosphopeptide enrichment protocols, an essential step in modern phosphoproteomic studies [24]. We used this TiO_2_ property [25] to develop a simple enrichment method (schematized in figure 1*a*) to extract inositol phosphates from acidic solutions, normally 1 M PA [19]. Initially, a specific amount of InsP_6_ diluted in PA was incubated with TiO_2_ beads for 30 min. After two washes with PA, InsP_6_ was eluted from the beads by a pH change induced by 10% ammonium hydroxide. After removing the ammonium hydroxide and reducing the volume using a centrifugal evaporator, the samples were resolved by PAGE and visualized with toluidine blue staining, demonstrating an almost complete recovery of the input InsP_6_ (figure 1*b*). We next tested this procedure on a *D. discoideum* extract, and recovered quantifiable levels of InsP_6_ and its pyrophosphate derivatives InsP_7_ and InsP_8_ (figure 1*c*). To precisely quantify the inositol phosphate recovery, radioactive ^3^H-Ins(1,4,5)P_3_ and ^3^H-InsP_6_ tracers were each mixed with 2 nmol of Ins(1,4,5)P_3_ and InsP_6_ and subjected to TiO_2_ enrichment. These experiments demonstrated that 87 ± 4.6 and 84 ± 3.5% (average ± s.d., *n* = 4) for Ins(1,4,5)P_3_ and InsP_6_, respectively, of input radioactivity was recovered after TiO_2_ elution, while about 2–4% remained attached to the TiO_2_ beads (figure 1*d*). The TiO_2_ beads are in fact completely efficient at binding and releasing inositol phosphates; the small loss is intrinsic to the manual handling involved.

### 4.2. Titanium dioxide purifies inositol phosphates and nucleotides from mammalian cell extracts

The lower concentration of inositol phosphates in mammalian cells has previously rendered extracts from these cells impracticable for PAGE analysis. Either the volume is too large to load onto the gel, or volume reduction by evaporation results in salt concentrations that cause aberrant gel migration. The ability to concentrate inositol phosphates using TiO_2_ beads overcomes these limitations.

**Figure 2.**
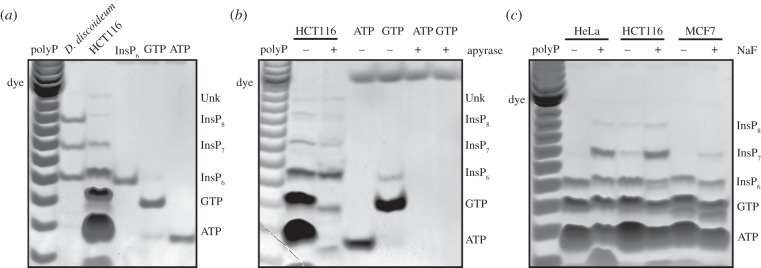
TiO_2_ beads purify nucleotides and inositol phosphates from mammalian cells. (*a*) PA extracts from vegetative *D. discoideum* (4 × 10^6^ cells) and the human HCT116 cell line (80 × 10^6^ cells) were subjected to TiO_2_ enrichment. After resolving the extract with PAGE, phosphate-rich molecules were visualized by toluidine blue staining for comparison with InsP_6_, ATP and GTP nucleotide standards. (*b*) TiO_2_-purified HCT116 extract and nucleotide standards were subjected to apyrase treatment, before resolution by PAGE and staining with toluidine blue. (*c*) Two 14 cm dishes of 80% confluent HeLa, HCT116 and MCF7 cells were treated with 10 mM sodium fluoride (NaF) for 1 h, before purification of inositol phosphates with TiO_2_ beads and resolution by PAGE with toluidine blue staining. The gels presented are representative of experiments performed at least three times.

**Figure 3.**
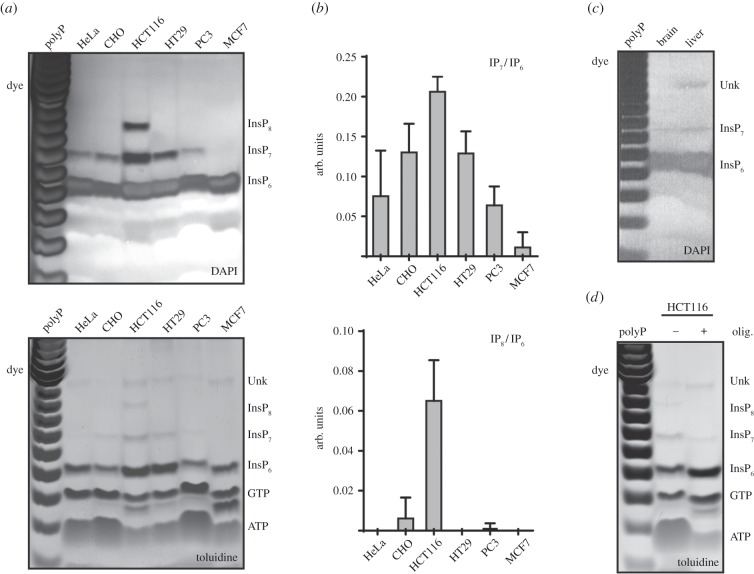
Visualizing InsP_6_, InsP_7_ and InsP_8_ from mammalian cells and organs. (*a*) Cells from six different human lines were collected and washed in PBS. A small aliquot was taken for determination of protein concentration, while the rest was PA extracted and subjected to TiO_2_ bead enrichment. Extracts relative to equivalent amounts of protein for each cell line (approx. 35 mg) were loaded on two parallel gels subsequently stained by DAPI (top) and toluidine blue (bottom). (*b*) Densitometry of toluidine blue-stained gel from three independent experiments was used to calculate ratios of InsP_7_ and InsP_8_ over their precursor InsP_6_. (*c*) Mouse brain and liver (approximately 0.5 g) were homogenized and extracted with PA. After TiO_2_ purification the inositol phosphates were resolved by PAGE and stained with DAPI. (*d*) Two 14 cm dishes of 80% confluent HCT116 cells were pre-treated in glucose-free medium for 30 min before addition of 5 µM oligomycin for 3 h. The TiO_2_ extracts were then resolved by PAGE and stained with toluidine blue. The gels presented are representative of experiments performed three times.

We tested TiO_2_ enrichment on extracts from HCT116 cells (human colon cancer cell line). PAGE analysis (figure 2*a*) of the phosphate-rich molecules extracted revealed the presence of three inositol phosphate bands that co-migrate with *D. discoideum*-extracted InsP_6_, InsP_7_ and InsP_8_ [10]. Interestingly, unlike *D. discoideum* extracts, mammalian cell extracts revealed extra bands that co-migrate with the nucleotide standards ATP and GTP. We confirmed the identity of the bands presumed to be ATP and GTP by treating the TiO_2_-purified samples with apyrase, an enzyme that specifically hydrolyses nucleotides (figure 2*b*). We also detected a faint, slower migrating band of unknown nature (labelled Unk), which is particularly abundant in liver extract (figure 3*c*). This band does not represent an InsP_9_ species since it is not fully degraded after phytase treatment unlike the InsP_6_, InsP_7_ and InsP_8_ bands (data not shown). The partial action of phytase on this unknown band suggests a complex molecule containing an inositol phosphate group. Inositol pyrophosphates in mammalian cells are known to be dramatically regulated by sodium fluoride (NaF) [26]. To confirm the identity of the observed InsP_7_ and InsP_8_ bands, we incubated three human cell lines with NaF: firstly, MCF7 cells, which usually have undetectable levels of inositol pyrophosphates; secondly, HeLa cells, which have detectable levels of InsP_7_; and thirdly, HCT116 cells, which have detectable levels of InsP_7_ and InsP_8_. PAGE analysis after TiO_2_ extraction revealed that NaF treatment increases inositol pyrophosphate levels, and decreased the level of their precursor InsP_6_, in all three cell lines (figure 2*c*).

### 4.3. Screening of mammalian cell lines and tissues for the presence of inositol pyrophosphates

Next, we decided to screen 27 mammalian, one plant and one *Drosophila* cell line for the presence of inositol pyrophosphates, to identify the best cell line(s) for studying the different aspects of inositol pyrophosphate metabolism. TiO_2_ purification was performed from 90% confluent cells grown in two 14 cm adherent culture dishes or shaking culture, as appropriate. The use of DAPI to better visualize inositol pyrophosphates [20] revealed the presence of InsP_7_ in almost all cells analysed (electronic supplementary material, figure S1), while InsP_8_ is easily detectable in mouse ES cells, *Drosophila* S2 and the human HCT116 cell line, where inositol pyrophosphates seem to be particularly abundant. An exact comparative analysis cannot be achieved since cell density, size and shape differ greatly between cell lines. Furthermore, DAPI staining is not linear, unlike toluidine blue, the staining intensity of which depends only on the number of phosphates [10]. Therefore, we chose several human cell lines to investigate their relative inositol pyrophosphate levels more thoroughly, normalizing the different extracts by protein mass. The normalized analysis of six mammalian cell lines by DAPI confirmed the screening result (figure 3*a*). Parallel toluidine blue staining confirmed a variable amount of InsP_7_ and especially InsP_8_ between the cell lines (figure 3*a,b*).

Coupling the TiO_2_ method with PAGE analysis also allows extraction and investigation of inositol pyrophosphates from previously intractable sources, including animal organs such as mouse brain or liver, where InsP_6_ and InsP_7_ can be easily detected (figure 3*c*).

### 4.4. Effect of altered energetic metabolism on inositol pyrophosphates levels

The ability to enrich and analyse InsP_7_ and InsP_8_ from mammalian extracts has the potential to revolutionize this field of research. As inositol pyrophosphates have been linked to cellular and organismal metabolism [27–29], we took advantage of the TiO_2_ method to observe their changes after metabolic perturbation. Inositol pyrophosphates were TiO_2_-extracted from glucose-starved HCT116 cells treated with the oxidative phosphorylation inhibitor oligomycin for 3 h. PAGE analysis showed the disappearance of InsP_8_ and a substantial reduction in InsP_7_, with a concomitant increase in InsP_6_ (figure 3*d*).

### 4.5. Absence of InsP_6_ in human blood revealed by titanium dioxide extraction

**Figure 4.**
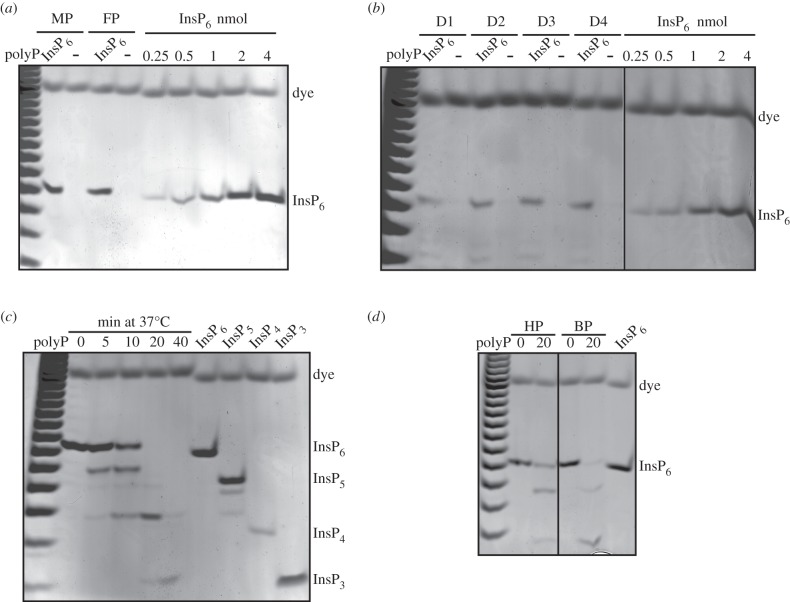
Absence of InsP_6_ and presence of inositol phosphate phosphatase in human plasma. All the extracts were resolved by PAGE and visualized with toluidine blue staining. (*a*) EDTA was added to 20 ml of commercial human plasma from male (MP) and female (FP); InsP_6_ was added to the spiked aliquot (InsP_6_; 2 nmol). The samples were then acidified and subjected to TiO_2_ enrichment. (*b*) Plasma from healthy anonymous donors (D1 to D4) was prepared as described in Material and methods, with spiking (InsP_6_; 1 nmol), and subjected to TiO_2_ extraction. (*c*) 4 nmol of InsP_6_ was added to 1 ml of human plasma and incubated at 37°C for the indicated time before acidification and extraction of inositol phosphates with the TiO_2_ procedure. Standards: InsP_6_ (4 nmol); InsP_5_ (6 nmol of Ins(1,3,4,5,6)P_5_); InsP_4_ (5 nmol of Ins(1,4,5,6)P_4_); InsP_3_ (20 nmol of Ins(1,4,5)P_3_). (*d*) 4 nmol of InsP_6_ was added to 1 ml of a different source of human plasma (HP) and bovine plasma (BP) before incubation at 37°C for the indicated time, followed by acidification of the samples and TiO_2_ extraction. The gels presented are representative of experiments performed two to four times.

**Figure 5.**
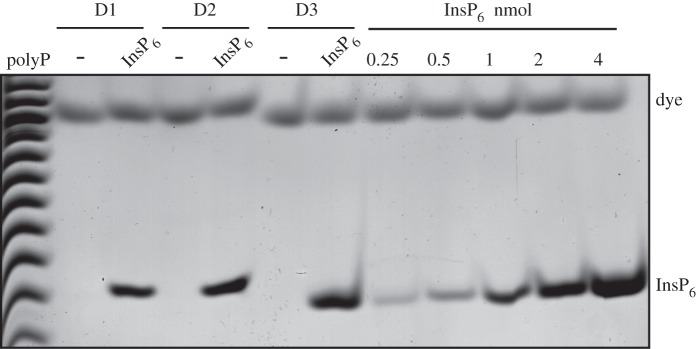
Absence of InsP_6_ in human urine. Freshly collected urine from healthy anonymous donors (D1–D3) was centrifuged to remove any epithelial cells. The samples were divided into two aliquots (25 ml each for D1 and D2, 10 ml for D3), EDTA was added and InsP_6_ (2 nmol) was supplemented into the spiked (InsP_6_) aliquots. The samples were then PA extracted and subjected to TiO_2_ enrichment. The extracted inositol phosphates were resolved by PAGE and visualized with toluidine blue staining. The gel presented is representative of three experimental repeats.

Since large volumes of acidified fluid can be subject to TiO_2_ bead extraction, this gave us the opportunity to assay InsP_6_ in biofluids. Initially, we used commercially available serum from bovine, equine and human sources. We extracted 20 ml of serum with TiO_2_ beads and analysed the extracts by PAGE. While we were able to detect an almost complete InsP_6_ recovery in the spiked samples, we did not recover any InsP_6_ in non-spiked serum (electronic supplementary material, figure S2A,B). We next analysed human plasma from a commercial source. Similar to serum, TiO_2_ extraction and PAGE analysis showed that InsP_6_ could not be recovered from non-spiked samples of human plasma (figure 4*a*). The lower limit of InsP_6_ standard detection on PAGE is about 0.25 nmol (figures 4*a*,*b* and 5), therefore TiO_2_-extracting 20 ml of plasma with a recovery of approximately 85% (figure 1*e*) indicates that the lower limit of plasma InsP_6_ we are able to extract and detect is approximately 15 nM. Consequently, we conclude that substantially less than 15 nM InsP_6_ is present in human plasma, in agreement with the enzymatic radio-assay previously reported [8].

Conversely, using less direct assays, others have suggested that the InsP_6_ concentration in human plasma is in the 0.2–0.5 µM range [13,14]. They suggested that the previous failure [8] to detect InsP_6_ in plasma was due to losses during plasma preparation [23]. To investigate this possibility, we here prepared plasma from anonymous donors following the extraction protocol described in [13,14] (which uses EDTA as an anti-coagulant [23]). While we were able to detect a good recovery of InsP_6_ from plasma when whole blood was spiked with 1 nmol (0.05 µM) of InsP_6_, we were again unable to detect any InsP_6_ in non-spiked human plasma (figure 4*b*). Interestingly, in the spiked samples, other faster migrating bands of weak intensity, probably lower forms of InsP*_x_*, were detected besides InsP_6_, and we only recovered spiked InsP_6_ with a high efficiency if the blood was cooled on ice before spiking. These observations suggested the presence of a phosphatase activity in plasma. To test this directly, we incubated InsP_6_-spiked human plasma at 37°C. Just 5 min of incubation resulted in substantial conversion of InsP_6_ to InsP_5_ (figure 4*c*); 20 min led to the complete removal of InsP_6_, while after 40 min all the exogenous InsP_6_ was converted to InsP_3_ and even lower forms of inositol phosphates. It is likely that a recently reported secreted mammalian phosphatase, MINPP1 (multiple inositol polyphosphate phosphatase) [30], is responsible for the observed activity. To further confirm the presence of phosphatase activity in plasma, a different source of human plasma together with bovine plasma was tested for the presence of this enzymatic activity (figure 4*d*). InsP_6_ phosphatase activity was detectable in both cases, indicating that this activity may be a common characteristic of mammals.

### 4.6. Absence of InsP_6_ in human urine revealed by titanium dioxide extraction

Another human biofluid in which InsP_6_ has been contentiously reported is urine, with some literature indicating that it reaches 1–3 µM concentration [31]. Therefore, we processed 10–25 ml of urine from anonymous donors using TiO_2_ and visualized the extraction on PAGE. Similar to the serum and plasma experiments, we were unable to detect any InsP_6_ in non-spiked urine (figure 5). Thus, less than 12 nM InsP_6_ is present in human urine, a maximal value in accordance with the earlier report using a specific enzymatic assay [8].

## 5. Discussion

TiO_2_ beads and pre-packed columns are useful tools for the enrichment of phosphopeptides and have contributed hugely to the development and application of phosphoproteomic studies [24]. We have taken advantage of this TiO_2_ phosphate binding property [25] to develop a new inositol phosphate purification protocol. Crucially, it allows for the purification of inositol phosphates from large volumes of acidified extracts and so makes feasible the extraction/enrichment of the low concentration inositol phosphates in mammalian extracts.

The direct analysis of mammalian InsP_6_ and its pyrophosphate derivatives InsP_7_ and InsP_8_ by coupling TiO_2_ enrichment with PAGE analysis negates the requirement for HPLC and ^3^H-inositol labelling. This new procedure therefore simplifies inositol pyrophosphate analysis in particular and also solves the often forgotten but ever-present problem of determining the labelling time necessary for metabolic equilibrium. Among the new research possibilities opened up by TiO_2_ purification is the analysis of inositol phosphates from animal tissues. Animal welfare and monetary considerations made this previously troublesome, as it would require treating the live animal with ^3^H-inositol tracer; analysis of human tissues and fluids was completely unattainable. The method described permits the extraction and analysis of inositol phosphates from animal organs including but not limited to mouse brain or liver, where InsP_6_ and InsP_7_ can be detected, or chicken egg white, where InsP_5_ and InsP_6_ are particularly abundant (Saiardi laboratory, unpublished data).

The ability of InsP_3_ to bind to TiO_2_ indicates that lower phosphorylated inositol species can also be purified from biological samples. As these stain poorly by toluidine blue they cannot be quantified by gel electrophoresis, but mass assays for other inositol phosphates exist that, coupled with TiO_2_ purification, can be used in quantifying lower phosphorylated inositol species from biological specimens. Beyond the soluble inositol phosphates, it will be interesting to take advantage of this newly discovered ability of TiO_2_ to bind and purify phosphate groups attached to an inositol ring to develop new inositol lipid purification methods, since the current approaches are essentially adaptations of Folch's extraction method, developed more than 60 years ago [32].

The parallel analysis of *D. discoideum* and mammalian cell extracts revealed quite different patterns of extracted molecules. While InsP_6_, InsP_7_ and InsP_8_ are extracted from both amoeba and mammalian cells, the nucleotides ATP and GTP are only visible in the mammalian extract. A quantitative comparison between the two experimental models is inappropriate since the number of cells extracted and analysed is different (in figure 2, 4 × 10^6^ cells for *D. discoideum* were compared to 80 × 10^6^ human HCT116 cells). However, the relative proportion between inositol pyrophosphate and nucleotides is unquestionably different in the two experimental models analysed. Since inositol pyrophosphates are able to regulate energetic metabolism and specifically are inversely connected with ATP level [27], we might speculate that the high levels of inositol pyrophosphates present in *D. discoideum* are lowering nucleotide levels. This hypothesis is currently under investigation.

We have also taken advantage of the high efficiency of TiO_2_ extraction to independently re-address the question of whether human body fluids such as plasma and urine contain any InsP_6_ (the relevance of this question is discussed below). In contrast with some other groups (e.g. [13,14]), but in agreement with an earlier study that used a specific and sensitive enzyme-based InsP_6_ assay [8], we find that there is no InsP_6_ present, with a detection limit more than an order of magnitude below the levels claimed to be there by others. We have carefully controlled for InsP_6_ recovery using InsP_6_-spiked controls (including adding exogenous InsP_6_ to whole fresh blood), and it is important to stress that our discovery of a highly active phosphatase in human (and bovine) plasma, probably secreted MINPP1 [30], which hydrolyses any spiked InsP_6_ within minutes at 37°C, in itself rules out the possibility of any InsP_6_ being present in plasma *in vivo*. Eiseman *et al.* [33] reported that in rats the half-life of intravenously injected radiolabelled InsP_6_ is 8 min, suggesting that the presence of an active InsP_6_ phosphatase in plasma is not confined to humans and cattle. Given the potent ion-chelating power of InsP_6_, it actually makes evolutionary sense to immediately remove such a compound (which might be released by cell lysis or damage) from extracellular fluids. We should add that the available evidence suggests that ingested InsP_6_, if absorbed through the gut, enters the blood plasma exclusively as inositol [33], with possibly also some small amounts of inositol monophosphate [34]; the dephosphorylation of InsP_6_ before absorption is apparently caused by gut flora [35].

These observations have relevance for the reported effects of dietary InsP_6_ on, for example, kidney stone formation [36,37] and other calcifications [38], or on cancer growth [39]. Understanding these effects does not need to invoke InsP_6_ in extracellular fluids, as they can readily be explained either by InsP_6_ acting as a chelator of cations (e.g. Ca^2+^, Fe^3+^) in the gut and thus altering uptake [8], or because InsP_6_ is a major source of our dietary inositol [8]. In the latter context, in studies on cancer where inositol has been compared directly with InsP_6_, the two have similar efficacies (e.g. [40] and see [39] for other references).

This discussion then points to a key question: if all the effects of dietary InsP_6_ (other than as a source of inositol) are mediated by modulating cation absorption from the gut, could taking InsP_6_ supplements ever be harmful? In humans on a poor diet the answer is clearly ‘yes’ [41], and there is a significant effort in the plant breeding world to produce low InsP_6_ varieties of maize and rice to reduce these deleterious effects [42]. Shamshuddin [43] has argued that the ion-chelating effect of InsP_6_ in the gut is not harmful in well-fed individuals, but this has only been examined for a few divalent cations (e.g. Zn^2+^ and Cu^2+^ [44,45]), while trivalent metals, whose affinity for InsP_6_ is very much higher than divalents [46] and which are essential dietary components (e.g. Cr^3+^ [47]), have not been studied in this context. Overall, this leads us to the conclusion that chronically altering cation absorption from the gut by artificially loading the diet with a non-specific chelator [39] in the hope that it might impact indirectly on cancer or other pathologies seems highly inadvisable.

## Supplementary Material

Supplementary Figures and Legends
