# Supplementary material for: A novel method for the purification of inositol phosphates from biological samples reveals that no phytate is present in human plasma or urine
Source: Open Biol. 2015 Mar 25;5(3):150014. doi: 10.1098/rsob.150014 (PMC4389793; doi:10.1098/rsob.150014)
Supplement: Supplementary Figures and Legends [file rsob150014supp1.pdf]

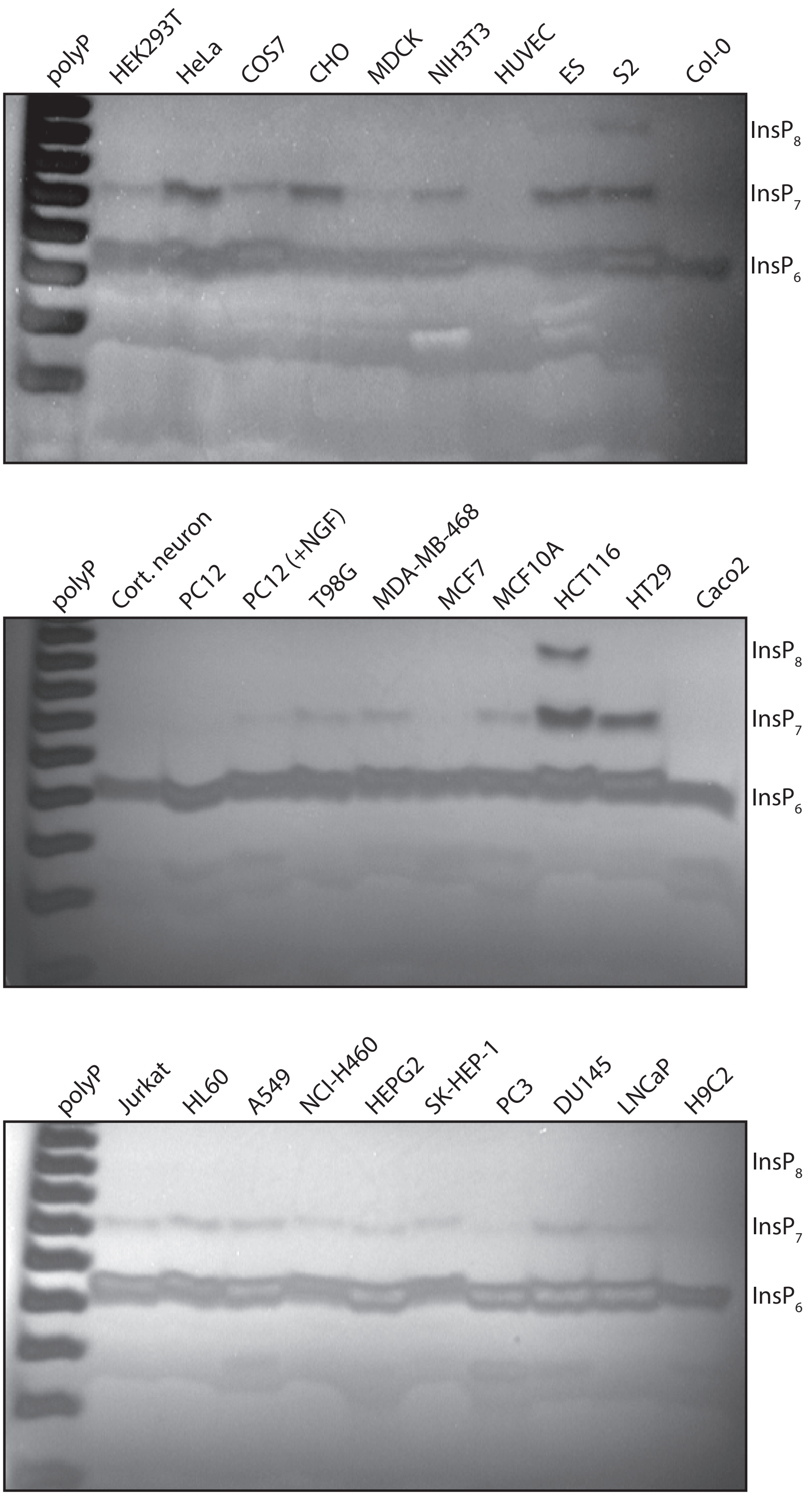

Supplementary Figure 1: Cell line screening for the presence of inositol pyrophosphates.
(A) Twenty-nine different cell lines were harvested, extracted with perchloric acid, and subjected to TiO_2 purification. The purified samples were resolved by PAGE and the inositol pyrophosphates revealed by DAPI photo-bleaching. Two 80-90 % confluent 14 cm dishes were used for each cell type, or equivalent for cells in shaking culture. All lines are immortal aside from HUVEC and rat primary cortical neuron. ES cells = mouse E14tg2a, passage 19. S2 cells are Drosophila. Col-0 cells are Arabidopsis. Cell amounts were not equal except for PC12 and PC12+NGF (5-day differentiated with 100 ng/ml NGF) which were normalised by protein mass (~10 mg each). The absence of inositol pyrophosphates in the primary cells HUVEC and rat cortical neurons likely reflects the smaller cell mass extracted compared to some of the immortal cell lines.

A

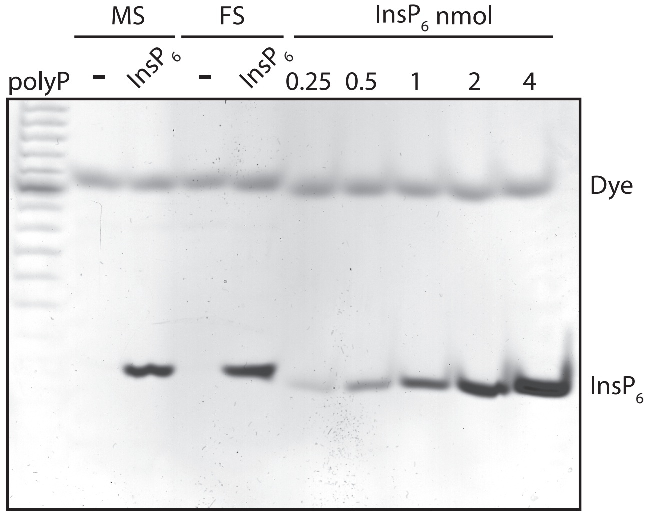

B

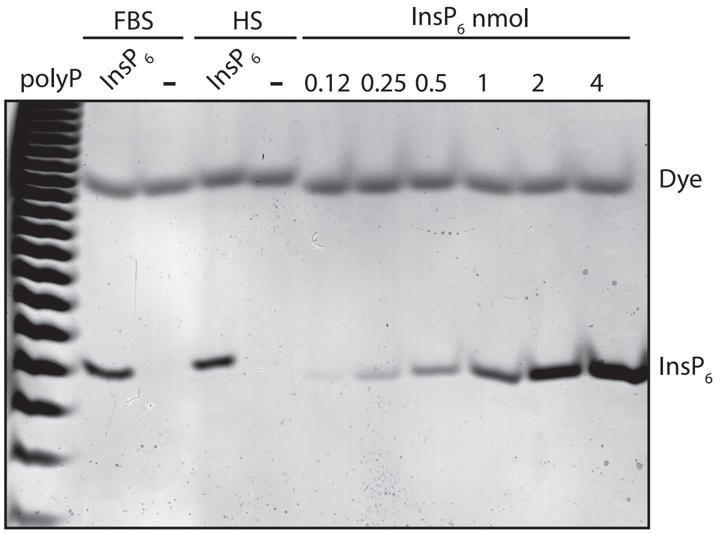

Supplementary Figure 2: Absence of $InsP_6$ in mammalian serum.
(A) EDTA was added to 20 ml of human serum from male (MS) or female (FS), with 2 nmol $InsP_6$ added to the spiked aliquot ($InsP_6$). The samples were then acidified and subjected to TiO_2 enrichment. (B) EDTA was added to 20 ml of foetal bovine serum (FBS) or horse serum (HS) with 1 nmol $InsP_6$ added to the spiked aliquot ($InsP_6$). The samples were then acidified and subjected to TiO_2 enrichment. All the extracts were resolved by PAGE and visualised with toluidine blue staining. The gels presented are representative of experiments performed three times.
